# The adenovirus major core protein VII is dispensable for virion assembly but is essential for lytic infection

**DOI:** 10.1371/journal.ppat.1006455

**Published:** 2017-06-19

**Authors:** Philomena Ostapchuk, Maarit Suomalainen, Yueting Zheng, Karin Boucke, Urs F. Greber, Patrick Hearing

**Affiliations:** 1 Department of Molecular Genetics and Microbiology, School of Medicine, Stony Brook University, Stony Brook, NY, United States of America; 2 Institute of Molecular Life Sciences, University of Zurich, Zurich, Switzerland; University of Michigan, UNITED STATES

## Abstract

The Adenovirus (Ad) genome within the capsid is tightly associated with a virus-encoded, histone-like core protein—protein VII. Two other Ad core proteins, V and X/μ, also are located within the virion and are loosely associated with viral DNA. Core protein VII remains associated with the Ad genome during the early phase of infection. It is not known if naked Ad DNA is packaged into the capsid, as with dsDNA bacteriophage and herpesviruses, followed by the encapsidation of viral core proteins, or if a unique packaging mechanism exists with Ad where a DNA-protein complex is simultaneously packaged into the virion. The latter model would require an entirely new molecular mechanism for packaging compared to known viral packaging motors. We characterized a virus with a conditional knockout of core protein VII. Remarkably, virus particles were assembled efficiently in the absence of protein VII. No changes in protein composition were evident with VII^−^virus particles, including the abundance of core protein V, but changes in the proteolytic processing of some capsid proteins were evident. Virus particles that lack protein VII enter the cell, but incoming virions did not escape efficiently from endosomes. This greatly diminished all subsequent aspects of the infectious cycle. These results reveal that the Ad major core protein VII is not required to condense viral DNA within the capsid, but rather plays an unexpected role during virus maturation and the early stages of infection. These results establish a new paradigm pertaining to the Ad assembly mechanism and reveal a new and important role of protein VII in early stages of infection.

## Author summary

The Ad major core protein VII protects the viral genome from recognition by a cellular DNA damage response during the early stages of infection and alters cellular chromatin to block innate signaling mechanisms. The packaging of the Ad genome into the capsid is thought to follow the paradigm of dsDNA bacteriophage where viral DNA is inserted into a preassembled capsid using a packaging motor. How this process occurs if Ad packages a DNA-core protein complex is unknown. We analyzed an Ad mutant that lacks core protein VII and demonstrated that virus assembly and DNA packaging takes place normally, but that the mutant is deficient in the maturation of several capsid proteins and displays a defect in the escape of virions from the endosome. These results have profound implications for the Ad assembly mechanism and for the role of protein VII during infection.

## Introduction

Adenovirus (Ad) infection is generally associated with mild respiratory, ocular, or gastrointestinal diseases, but Ad has been recognized in recent years as a significant pathogen in immunocompromised patients and in the young and elderly [1]. Ad replication involves a number of events that must be temporally and spatially organized within the host cell in order to lead to optimal productive infection. The 36-kbp Ad genome encodes at least 25 early gene products and ~15 late gene products. The early viral proteins alter host cell functions to promote an environment that is conducive to viral replication and to block cellular and host antiviral responses, as well as for the enzymatic replication of the Ad genome [2]. The late gene products comprise structural components of the capsid, as well as proteins involved in virion assembly and maturation and viral genome encapsidation [3]. Ad is an excellent example of a virus that efficiently utilizes limited genetic capacity to maximize viral protein and virion production.

Viral nucleic acids are sensed following infection by pathogen recognition receptors, and other cytoplasmic and nuclear effectors, to trigger cellular, antiviral responses [4]. With Ad, viral infection triggers the cGAS/STING pathway and activation of type I interferon (IFN) signaling [5]. Recent studies have shown that cGAS/STING activation stimulates the TBK1/IRF3 cascade to promote type I IFN production and the activation of IFN-stimulated genes (ISG)[6]. One such ISG that is known to promote both intrinsic and innate responses to viral infection is the product of the promyelocytic leukemia (PML) gene [7]. The PML protein nucleates the formation of PML nuclear bodies (PML-NB) that exert antiviral effects on a wide range of viruses. Many viruses, including Ad, express proteins that interfere with PML-NB activity. Ad utilizes different mechanisms to counteract IFN signaling pathways. These include E1A inhibition of different aspects of IFN signaling [4, 8–10], E1B-55K inhibition of ISG expression and function [11–13], inhibition of STAT1 phosphorylation and nuclear translocation by the E3-14.7K protein [14], sequestration of nuclear STAT1 into viral replication centers [15], and Ad VA RNA-I inactivation of PKR [16].

Viruses with linear, dsDNA genomes, such as Ad, also trigger a cellular DNA damage response (DDR)[17]. With Ad, the DDR severely inhibits viral DNA replication if unabated [18]. Ad has evolved two redundant mechanisms to inhibit a DDR, involving the E4-ORF3 protein and the E1B-55K/E4-ORF6 protein complex, to allow efficient Ad DNA replication to occur. With both mechanisms, Ad proteins target and inhibit the sensors of DNA damage, the Mre11-Rad50-Nbs1 (MRN) complex [18]. Our results support the hypothesis that the major Ad core protein, protein VII, protects the viral genome from recognition by a DDR during the early stages of infection until the E1B and E4 gene products are synthesized to counteract this response [19]. Ad core protein VII also impacts innate cellular signaling mechanisms. A recent study demonstrated that Ad core protein VII binds to and regulates cellular chromatin and sequesters immune danger signals to control immune signaling [20].

The Ad genome within the virion is associated with ~500–800 copies of core protein VII [21, 22]. Core protein VII condenses DNA *in vitro* and *in vivo* and assembles viral DNA into a nucleosome-like structure [23–28]. It has been widely assumed that protein VII is required to condense viral DNA within the Ad capsid [29]. Core protein VII remains associated with the Ad genome during the early phase of virus infection [30–32] and is released from viral DNA coincident with early gene transcription [31]. Human Ads also encode two other basic core proteins, proteins V and X/μ, which are packaged into the virion [33]. Several lines of evidence suggest that the Ad genome is positioned within the capsid in globular domains [34, 35], but the structure of the Ad DNA-protein core is unknown [29]. Protein V of the incoming virions remains in the cytoplasm following escape of the Ad core from the endosome [36]; the fate of core protein X/μ during virus disassembly has not been determined.

The packaging of the Ad genome into the capsid is thought to follow the paradigm of dsDNA bacteriophage assembly where viral DNA is inserted into a preformed, empty capsid using a packaging motor [37]. Highly sophisticated studies have been conducted on the assembly of dsDNA bacteriophage [38]. A precursor viral capsid, the prohead, is formed that contains a unique portal vertex through which naked viral DNA is packaged. A portal protein complex is found at this vertex which associates with a powerful packaging motor, the terminase. The terminase contains a DNA translocation ATPase and a concatamer-resolving endonuclease. The DNA packaging motor uses the hydrolysis of ATP to translocate DNA into the capsid [39]. Within the capsid, the viral DNA associates with protamines and cations to neutralize the negative charge and allow genome compaction. A key feature of this packaging process is that there is a direct electrostatic interaction between the phosphate backbone of naked viral DNA with the ATPase subunits of the packaging motor to direct DNA packaging in a stepwise process [40]. An Ad assembly intermediate has been identified that corresponds to a prohead and several lines of evidence suggest that this virus particle is a precursor for viral DNA packaging [41, 42]. The Ad IVa2 protein is present at a unique vertex [43] and contains conserved Walker A and B box motifs that are hallmarks of ATPases. The IVa2 protein binds ATP *in vitro* and the Walker A and B box motifs are required for ATP binding and for viral DNA encapsidation [44]. It is not known if naked Ad DNA is packaged, as with bacteriophage, followed by the encapsidation of viral core proteins, or if a unique packaging mechanism exists with Ad where a DNA-protein complex is simultaneously packaged into the capsid [37]. The latter model would require an entirely new molecular mechanism for viral DNA packaging compared to known bacteriophage packaging motors. If naked Ad DNA is packaged and core proteins inserted into the capsid following genome encapsidation, then another packaging process must be proposed to account for the insertion of core proteins into the capsid. An alternative view of Ad virion assembly is a concerted model where capsids assemble around a viral DNA-protein core [45].

Here, we studied the role of the Ad major core protein VII in the viral life cycle. Our results demonstrate that protein VII is not required for virion assembly or Ad genome packaging, but rather protein VII plays a critical role in virus escape from the endosome following infection. Thus, Ad core protein VII plays an unexpected role during early stages of virion entry. These results establish new paradigms pertaining to the Ad assembly mechanism and the role of core protein VII during infection.

## Results

### Conditional expression of Ad5 core protein VII

**Fig 1:**
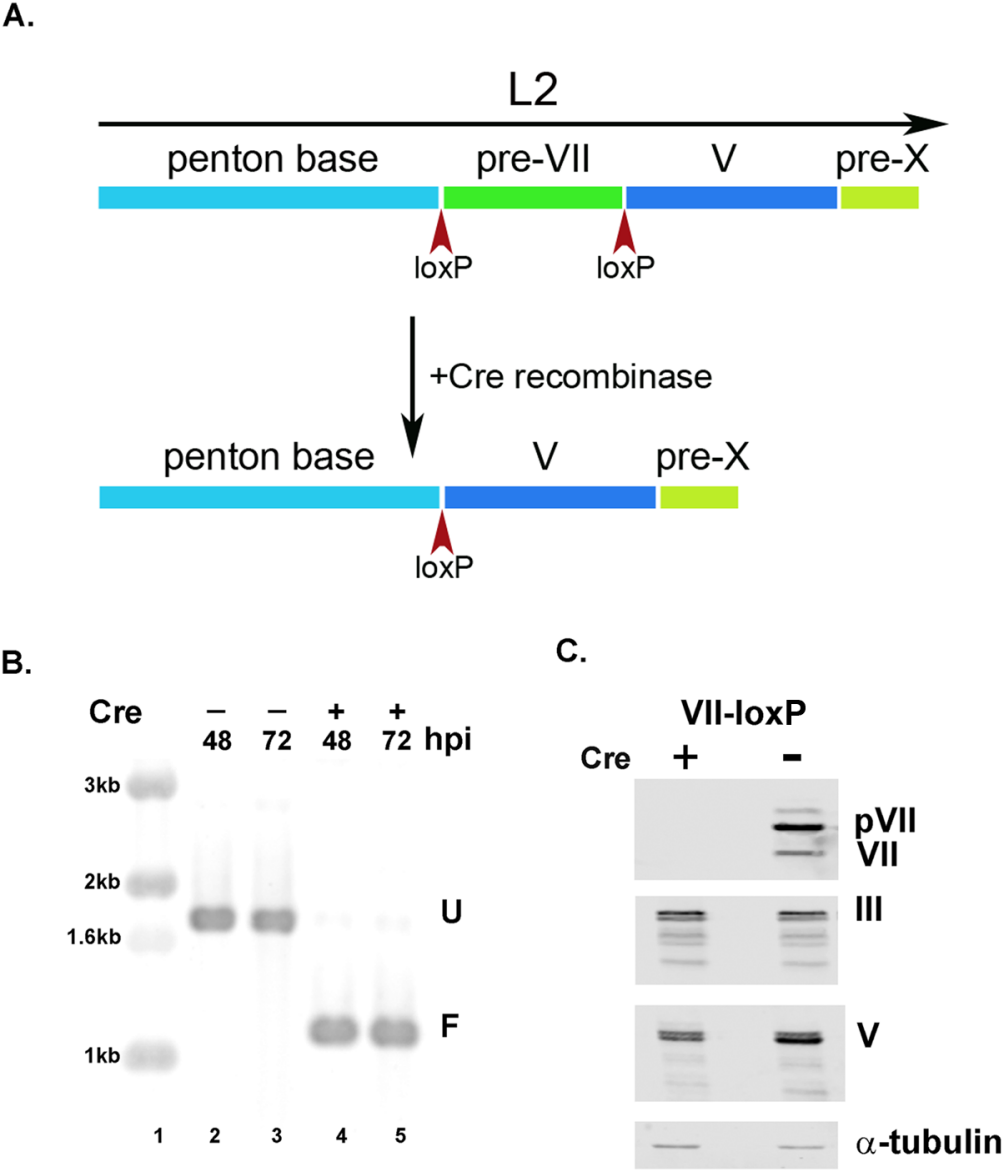
Cre-mediated recombination of the pVII gene from the Ad5-VII-loxP viral genome. A) Schematic of the Ad5 L2 coding region. Open reading frames that encode the proteins penton (III), pVII, V, and X/μ are depicted in colors. LoxP sites are indicated by upward red arrows. The downward arrow indicates infection of a Cre recombinase-expressing cell line with Ad5-VII-loxP. Cre-mediated recombination leads to excision of the pVII gene with the maintenance of a single lox site. (B) Southern blot analysis of total cell DNA isolated from infections of either 293 cells (–, lanes 2 and 3) or 293 cells expressing Cre recombinase (+, lanes 4 and 5) with the Ad5-VII-loxP virus. Cells were harvested at 48 (lanes 2 and 4) or 72 (lanes 3 and 5) hr post-infection (hpi) and DNAs were analyzed by Southern blot. U represents intact (unfloxed) pVII gene and F represents the recombined (floxed) pVII gene. DNA size markers are indicated on the left (lane 1). (C) Western blot analyses of lysates from 293 cells (–) and 293 cells expressing Cre (+) infected with the Ad5-VII-loxP virus. Ad5 L2 proteins were detected with specific antibodies as described in Supplemental Information. Cellular alpha-tubulin (α-tubulin) was used as a loading control.

The Ad5 L2 region (Fig 1A) encodes four viral proteins: penton base (capsid protein III) and the three core proteins pre-VII, V, and pre-X. The precursors of protein VII (pre-VII) and protein X (pre-X) are proteolytically processed by the Ad proteinase AVP to mature forms designated VII and μ, respectively [3]. We wanted to analyze the role of the major Ad core protein VII in virus assembly and other aspects of the viral life cycle. A traditional approach to analyze viral mutants is to establish a complementing cell line to allow the propagation of a defective virus. However, it has been difficult to produce cell lines that efficiently complement the growth of Ad late protein mutants, for example owing to the high levels of protein expression that are required for complementation and the potential toxicity of their expression to cells.

We established an approach to conditionally knock out the gene encoding pre-VII in the context of virus infection using the Cre-lox system. An Ad5 infectious clone was generated with loxP sites flanking the pre-VII coding sequences (Fig 1A; loxP sites at -4 relative to the A of the ATG and immediately following the pre-VII termination codon; the virus is termed Ad5-VII-loxP). Recombinant Ad5-VII-loxP virus stocks were readily established in cells that do not express Cre recombinase, and the virus replicated with wild-type kinetics as measured by qPCR. Two independent Ad5-VII-loxP virus stocks were analyzed (isolates 5 and 11). Ad5-pVII-loxP was used to infect cells that express Cre recombinase, and the efficiency of excision of pre-VII coding sequences (floxing) was measured by Southern blot (Fig 1B) and qPCR. Ad5-VII-loxP floxing efficiency was ≥99% in 293 cells that express Cre recombinase (lanes 2, 3 vs. 4, 5). We examined pre-VII/VII protein levels following infection of cells that do or do not express Cre recombinase with Ad5-VII-loxP by Western blot (Fig 1C). Infection of Cre recombinase-expressing cells with Ad5-pVII-loxP reduced pre-VII/VII protein expression to barely detectable levels in comparison to infection of parental 293 cells. Equivalent levels of L2 proteins III and V were detected in cells that do or not express Cre recombinase demonstrating a specific effect on pre-VII/VII protein expression and not a global deficit in L2 gene expression. These results establish the utility of using Cre recombinase to direct conditional pVII/VII protein expression.

### Core protein VII is not required for packaging of the viral genome into the capsid

**Fig 2:**
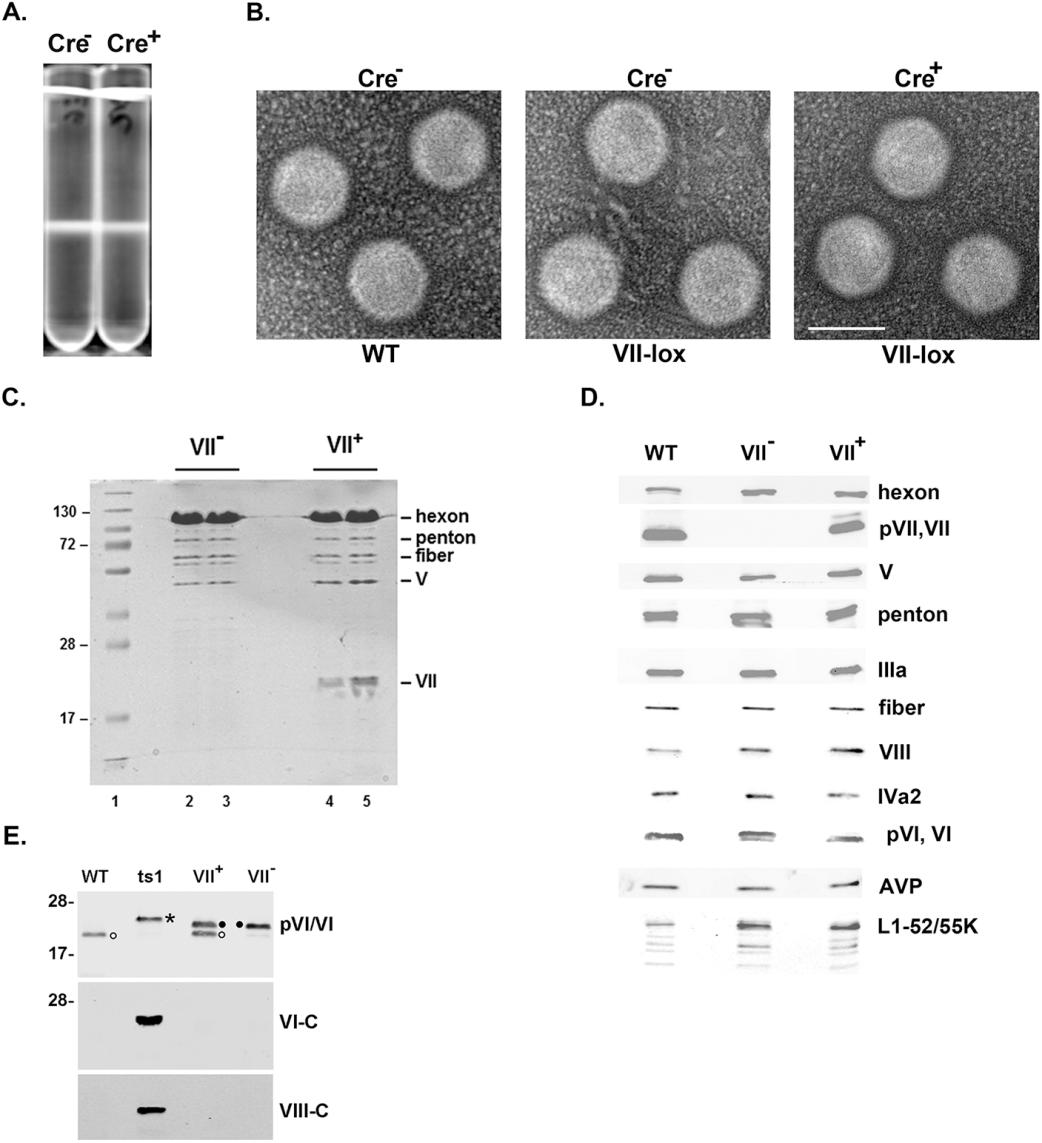
The absence of pVII does not preclude virus particle assembly and genome packaging. (A) CsCl equilibrium gradient centrifugation of virus particles isolated from isolated from 293 cells (Cre^–^) or 293 cells expressing Cre recombinase (Cre^+^) infected with the Ad5-VII-loxP virus. Virus particles from both infections banded on the gradient at 1.34 g/cc, the density of mature Ad5 virions. (B) Electron microscopy images of negative stained CsCl-purified virions isolated from 293 cells (Cre^–^) or 293 cells expressing Cre recombinase (Cre^+^) infected with wild-type Ad5 (WT) or the Ad5-VII-loxP virus. Bar inset represents 50nm. (C) Coomassie blue stain analysis of the protein composition of CsCl-purified virions isolated from 293 cells infected with wild-type Ad5 (WT) or the Ad5-VII-loxP virus (VII^+^) or 293 cells expressing Cre recombinase (VII^–^) infected with the Ad5-VII-loxP virus. Virus particles from the Ad5-VII-loxP-infected cells were harvested 48 and 72 hr post-infection (hpi). Protein molecular weight markers are indicated on the left. Major Ad capsid proteins are indicated on the right. (D) Western blot analyses of purified virus particles described in (C). Ad capsid proteins are indicated on the right. (E) Western blot analysis of purified virus particles described in (C), plus *ts*1 particles isolated following infection of cells at the restrictive temperature, probed with antibodies directed against pVI/VI, or C-terminal peptides of pVI (VI-C) or pVIII (VIII-C). The asterisk (*) indicates full-length, unprocessed pre-VI, the bullet (•) indicates iVI, and the open circle (o) indicates fully processed VI.

We examined the production of virus particles following infection of cells that do or do not express Cre recombinase with Ad5-VII-loxP. Ad5-VII-loxP virus particles were efficiently produced in cells that express Cre recombinase, and the virions produced in Cre-expressing or parental 293 cells banded at the same density in a CsCl equilibrium gradient (1.34 g/cc)(Fig 2A, isolate 5). Virus particles that banded at this density contained the full-length viral genome and Ad5-VII-loxP particles produced in Cre-expressing cells were floxed ≥99% (S1A and S1B Fig). These virus particles were examined by electron microscopy in comparison to wild-type Ad5 (Fig 2B). Virus particles that contained or lacked protein VII (VII-lox, Cre^+^, Cre^–^, respectively) were indistinguishable and had the same morphology and electron density as wild-type Ad5.

We examined virion protein composition by SDS-PAGE and Coomassie blue and silver staining and by Western blot. The identical pattern of major and minor viral capsid proteins were observed with the only detectable difference being the lack of protein VII in Ad5-VII-loxP particles produced in Cre-expressing cells (Figs 2C and S1C). By western blot, all Ad late major and minor capsid proteins were equally represented in Ad5-VII-loxP particles produced in Cre-expressing cells compared to parental 293 cells, and in comparison to wild-type Ad5, with the sole exception of protein VII (Fig 2D). We conclude that Ad5 protein VII is not required for packaging of the viral genome into the virion and that the loss of protein VII in the capsid is not compensated by an increase in the levels of core protein V or other capsid proteins.

We did note an effect on the proteolytic processing of Ad late protein pre-VI when protein VII was missing (Fig 2D, pre-VI, VI; doublet with VII^−^compared to a single form with WT). Pre-VI is proteolytically cleaved by AVP at both the N- and C-termini, whereas pre-VIII is processed at three internal cleavage sites [46]. The processing of proteins pre-VI and pre-VIII was examined further by western blot using specific antisera (Fig 2E). Ad2 temperature-sensitive mutant *ts*1 is defective for AVP at the restrictive temperature, accumulates precursor forms of Ad late proteins, and was used as a control [23]. *Ts*1 virions contained the unprocessed form of pre-VI (indicated by an asterisk in the top panel; pVI/VI) and pre-VIII (VIII-C). Mature VI, was observed with wild-type Ad5 virions (indicated by an open circle in the top panel). Virions that lacked protein VII contained a partially processed form of pre-VI (termed intermediate VI, iVI, [47]; indicated by a bullet in the top panel) that exhibited a faster mobility than *ts*1 pre-VI and that was processed at the C-terminus (VI-C), but presumably not cleaved by AVP at the N-terminus. Pre-VIII C-terminal processing in virions that lacked protein VII occurred normally (VIII-C). The parent Ad5-VII-loxP virions contained both iVI and mature VI. The basis for this observation is not clear; the genomic pre-VI region was fully sequenced with both infectious clone isolates of Ad5-VII-loxP and the sequences were wild-type.

### Virus genomes that lack core protein VII are defective in early gene expression and viral DNA replication

**Fig 3:**
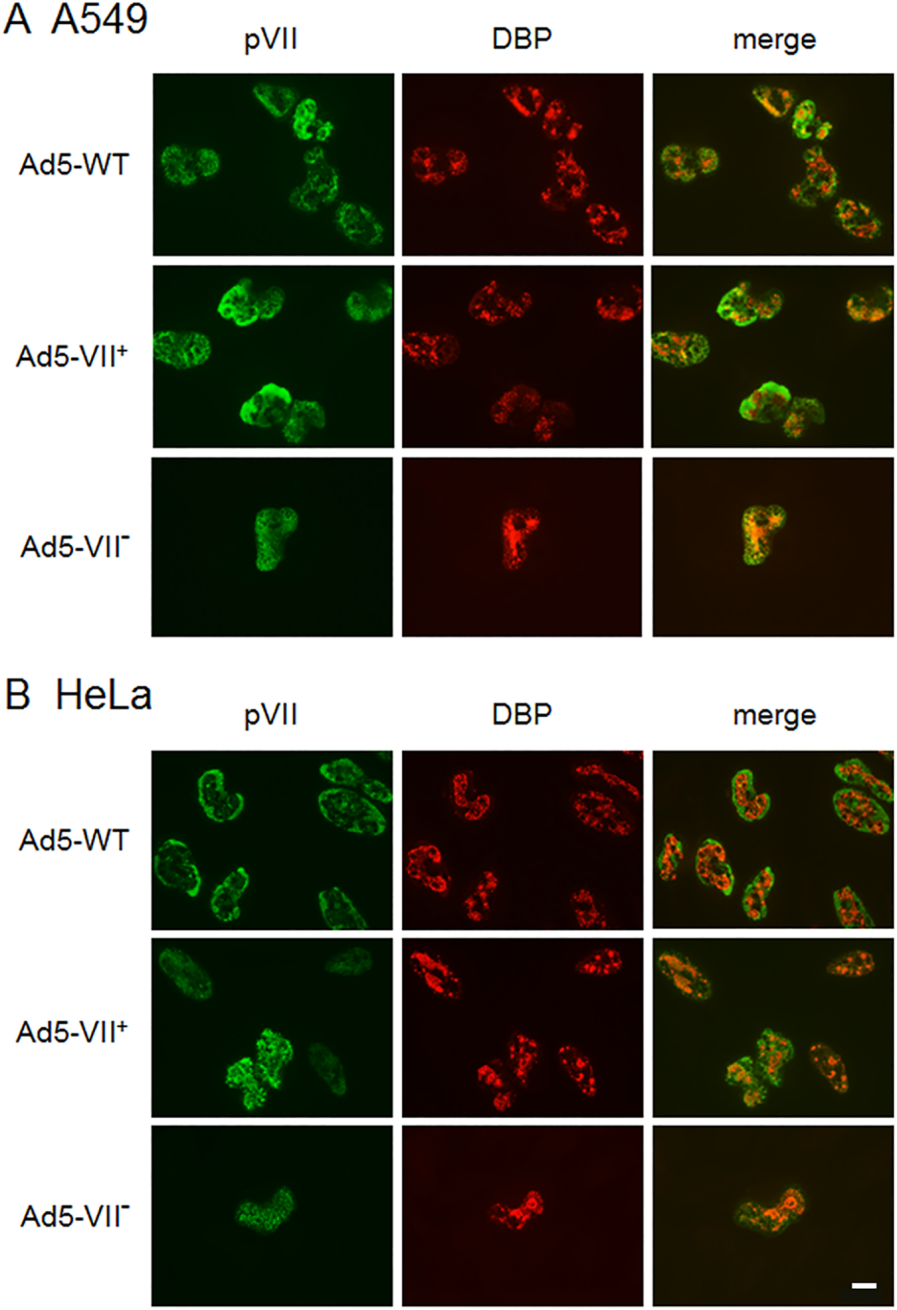
Infection of cells with virus particles that lack protein VII. A549 cells (A) or HeLa cells (B) on glass coverslips were infected with 200 particles/cell wild-type Ad5 or the Ad5-VII-loxP virus grown in 293 cells (Ad5-VII^+^), or 2000 particles/cell VII^−^virus (Ad5-VII^–^). At 24 hours post-infection, cells were processed for IF using antibodies directed against protein VII (FITC secondary antibody; left column) and Ad DNA binding protein (TRITC secondary antibody; center column). Merged images are shown in the right column. The white bar represents 10μM.

We examined the infectivity of virus particles that lack protein VII using a fluorescent focus assay in HeLa cells and found a >300-fold decrease in infectivity compared to wild-type Ad5 and a >100-fold decrease compared to Ad5-VII-loxP virus grown in 293 cells (Particle:FFU ratios were 5:1 for Ad5-WT, 13.5:1 for Ad5-VII-loxP grown in 293 cells, and 1820:1 for Ad5-VII-loxP grown in Cre-expressing cells). We analyzed the formation of viral replication centers in A549 and HeLa cells at low multiplicity of infection with wild-type Ad5 and Ad5-VII-loxP virus grown in 293 cells and at high multiplicity of infection with Ad5-VII-loxP virus grown in 293-Cre cells by immunofluorescence (IF) (Fig 3A, A549 cells, Fig 3B, HeLa cells). The presence of circular, DBP-positive viral replication centers in the nucleus directly correlates with active viral DNA replication. Viral replication centers were readily evident in cells infected at low multiplicity of infection with wild-type Ad5 or the VII^+^ virus. There was a striking reduction in the number of replication foci observed 24 hours after infection with virus that lacks protein VII where very few DBP-positive cells were evident even at high multiplicity of infection. Further, of the few viral replication centers that were observed following infection with VII^−^virus particles, all were found to also express protein VII (Fig 3A and 3B) indicating that these cells were infected with Ad5-VII-loxP virions that had escaped VII floxing during production. We also used IF to visualize protein VII expression in Cre-expressing cells infected with Ad5-VII-loxP. Very few Cre-expressing cells exhibited protein VII expression at late times after infection, whereas the Ad DNA binding protein was readily evident in all infected cells. Thus, we believe that a small percentage (≤1%) of the input Ad5-VII-loxP viral genomes escaped floxing in Cre recombinase-expressing cells, whereas the majority of the viral genomes (≥99%) were fully floxed. Based on this conclusion, the majority of Ad5-VII-loxP virus particles produced in Cre-expressing cells should be fully deficient for protein VII, while a small percentage (≤1%) contain the full complement of protein VII and are equivalent to wild-type Ad5.

**Fig 4:**
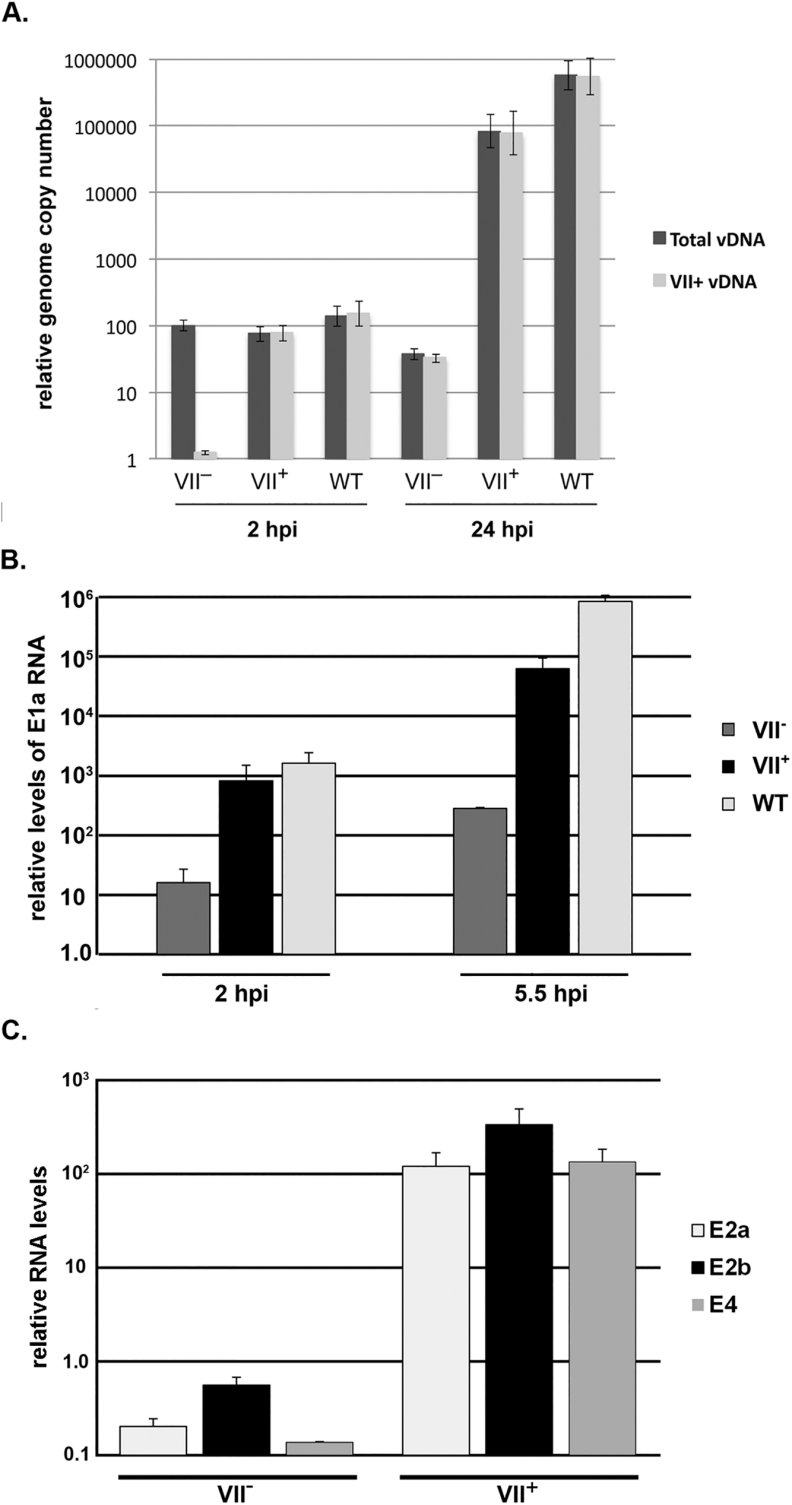
Core protein VII is required for efficient viral early gene expression and DNA replication. (A) HeLa cells were infected with wild-type Ad5 (WT) and the VII^+^ and VII^−^viruses, and virus infection (2 hpi) and viral DNA replication (24 hpi) was quantified by qPCR. Primer pairs for qPCR hybridized to all three viruses (Total vDNA) or were specific to the pVII open reading frame (VII+ vDNA). (B) HeLa cells were infected with wild-type Ad5 (WT) and the VII^+^ and VII^−^viruses and E1a mRNA levels were quantified by RT-qPCR at 2 and 5.5 hr post-infection (hpi). The values were normalized to cellular GAPDH mRNA levels as described in Materials and methods. (C) The VII^+^ and VII^−^viruses were used to infect 293 cells and E2a, E2b, and E4 mRNA levels were quantified by RT-qPCR at 5.5 hpi and normalized to cellular GAPDH mRNA levels. Values are plotted as mean ± sd.

The lack of core protein VII in the Ad5 particle could affect virus infectivity at a number of different steps during the viral life cycle. To determine the basis for the defect observed with the VII^−^mutant virus, we examined input viral DNA levels in infected cells at 2 hours post-infection (2 hpi) and viral DNA replication at 24 hpi by quantitative PCR using total genome and VII gene-specific primer pairs (Fig 4A). At 2 hpi, the level of VII^−^mutant viral DNA in total cell lysates was comparable to wild-type Ad5 and the unfloxed Ad5-VII-loxP parent virus; efficient floxing of the VII^−^mutant stock was verified (2 hpi, VII^–^, total vDNA versus VII^+^ vDNA). At 24 hpi, there was a 4-log decrease in the levels of VII^−^mutant DNA compared to wild-type Ad5 and a 3-log decrease compared to the unfloxed Ad5-VII-loxP parent virus. Further, the few genomes that replicated following infection with the VII^−^virions contained an intact VII reading frame and, thus, represented the low level of unfloxed viral genomes present in the VII^−^stock (24 hpi, VII^–^, total vDNA versus VII^+^ vDNA). These results are consistent with the IF data shown in Fig 3.

We tested if the decrease in viral DNA replication observed with the VII^−^virus resulted from reduced E1A gene expression, and found that the E1A mRNA levels were strongly reduced in cells infected with VII^−^virions (Fig 4B). In order to determine if this defect in early gene expression with the VII^−^mutant virus could be rescued if the E1A proteins were provided *in trans*, 293 cells, which constitutively express Ad5 E1A proteins, were infected with Ad5-VII-loxP produced in 293 cells (VII^+^) or Cre-expressing cells (VII^–^)(Fig 4C). RNA levels for Ad early regions E2a, E2b, and E4 were reduced >100-fold in 293 cells infected with the protein VII^−^mutant virus in comparison to the VII^+^ virus, indicating no rescue of viral early gene expression. Finally, we performed a coinfection experiment with wild-type Ad5 together with the VII^+^ or VII^−^viruses and examined viral DNA levels by qPCR at early and late times after infection (S2 Fig). Input levels of the coinfecting viral DNAs were similar early after infection (WT+VII^+^ vs WT+VII^–^, 3 hpi). Wild-type Ad5 and the VII^+^ virus replicated to similar levels by late times after infection, whereas the VII^−^mutant virus was significantly reduced (24 hpi). We conclude that the VII^−^mutant virus is defective in an early step in the infection cycle. Ad5 particles that lack core protein VII show a global defect in early gene expression and viral DNA replication that cannot be complemented *in trans*.

### VII^−^viral genomes are functional outside the context of virus infection

**Fig 5:**
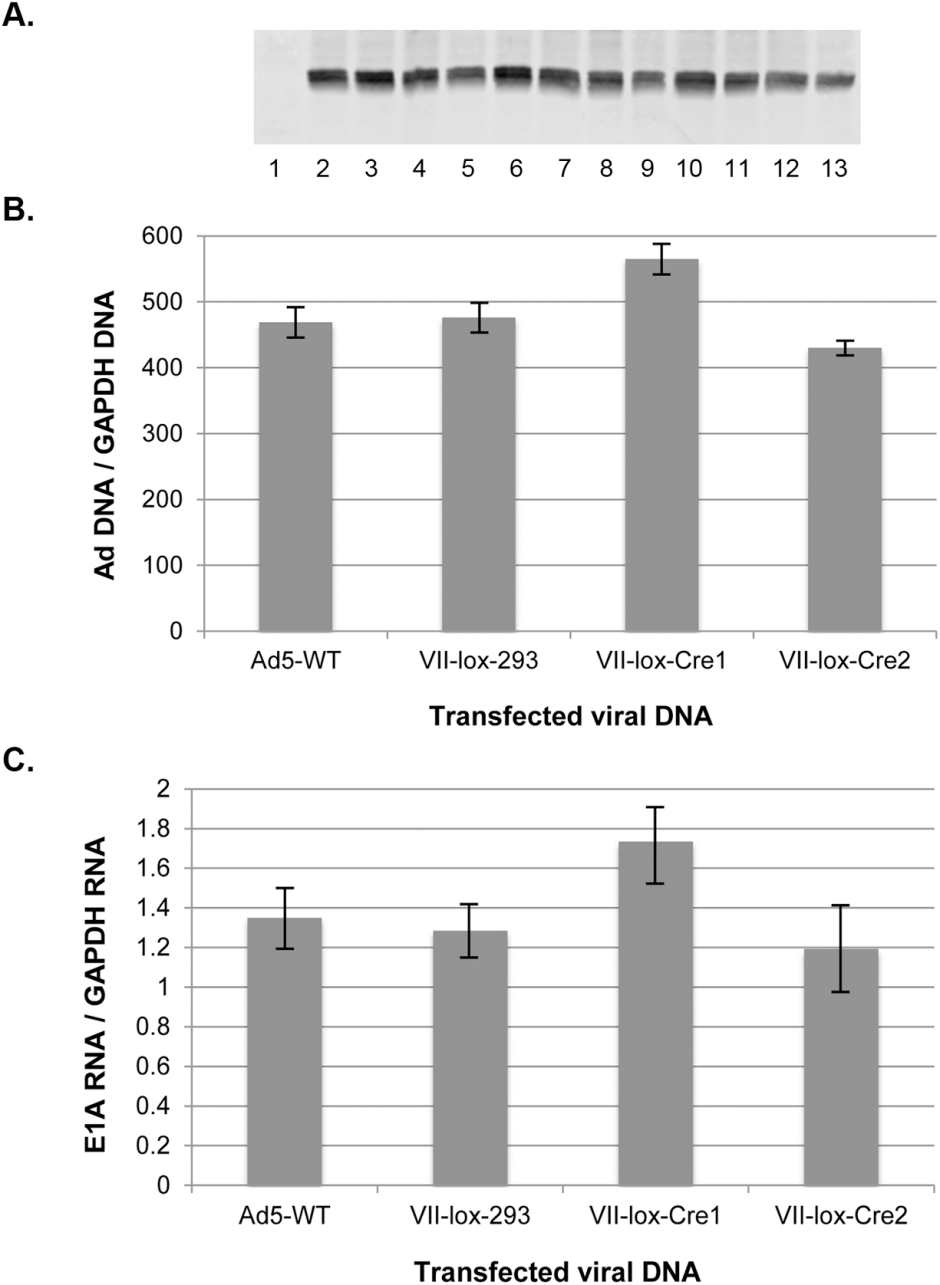
Transfected VII^–^ virion DNA fosters productive infection. HeLa cells were cotransfected with purified viral DNAs and an EGFP expression vector. Viral DNAs corresponded to Ad5-WT, Ad5-VII-loxP grown in 293 cells (VII-lox-293), and two independent preparations of Ad5-VII-loxP grown in 293-Cre cells (VII-lox-Cre1 and VII-lox-Cre2). 48 hours after transfection, the cells were harvested and used for the preparation of whole cells protein extracts, total cell DNA, and total cell RNA, as described in Materials and Methods. (A) EGFP Western blot of samples isolated from three independent transfection experiments. Lane 1, untransfected; lanes 2, 6, and 10, cells transfected with Ad5-WT DNA; lanes 3, 7, and 11, cells transfected with VII^+^ DNA; lanes 4, 8, and 12, cells transfected with VII^−^DNA preparation 1; lanes 5, 9, and 13, cells transfected with VII^−^DNA preparation 2. (B) Total cellular DNA isolated from these transfections were digested with DpnI to cut transfected, input plasmid DNA, and analyzed by qPCR for Ad5 DNA levels using a primer pair that PCRs across a segment with two DpnI sites. (C) Total cellular RNA was these transfections was analyzed for E1a mRNA levels as described in Fig 5B. n = 3 for (B) and (C) and the values are plotted as mean ± sd.

Viral DNAs isolated from VII^+^ and VII^−^virus particles were used to transfect cells, and viral DNA and E1A mRNA levels were analyzed by qPCR and RT-qPCR, respectively. An EGFP expression vector was used as a transfection control, and equivalent levels of GFP protein expression was observed in three replicate experiments (Fig 5A). No significant differences were observed between viral DNAs isolated from VII^+^ (Ad5-WT, VII-lox-293) or VII^−^(VII-lox-Cre1, VII-lox-Cre2) virus particles when viral DNA replication (Fig 5B) or E1A mRNA levels (Fig 5C) were examined. These results demonstrate that virion DNA in VII^−^particles is functional when used for transfection, but not in the context of viral infection.

### Protein VII is required for escape of virions from endosomes

**Fig 6:**
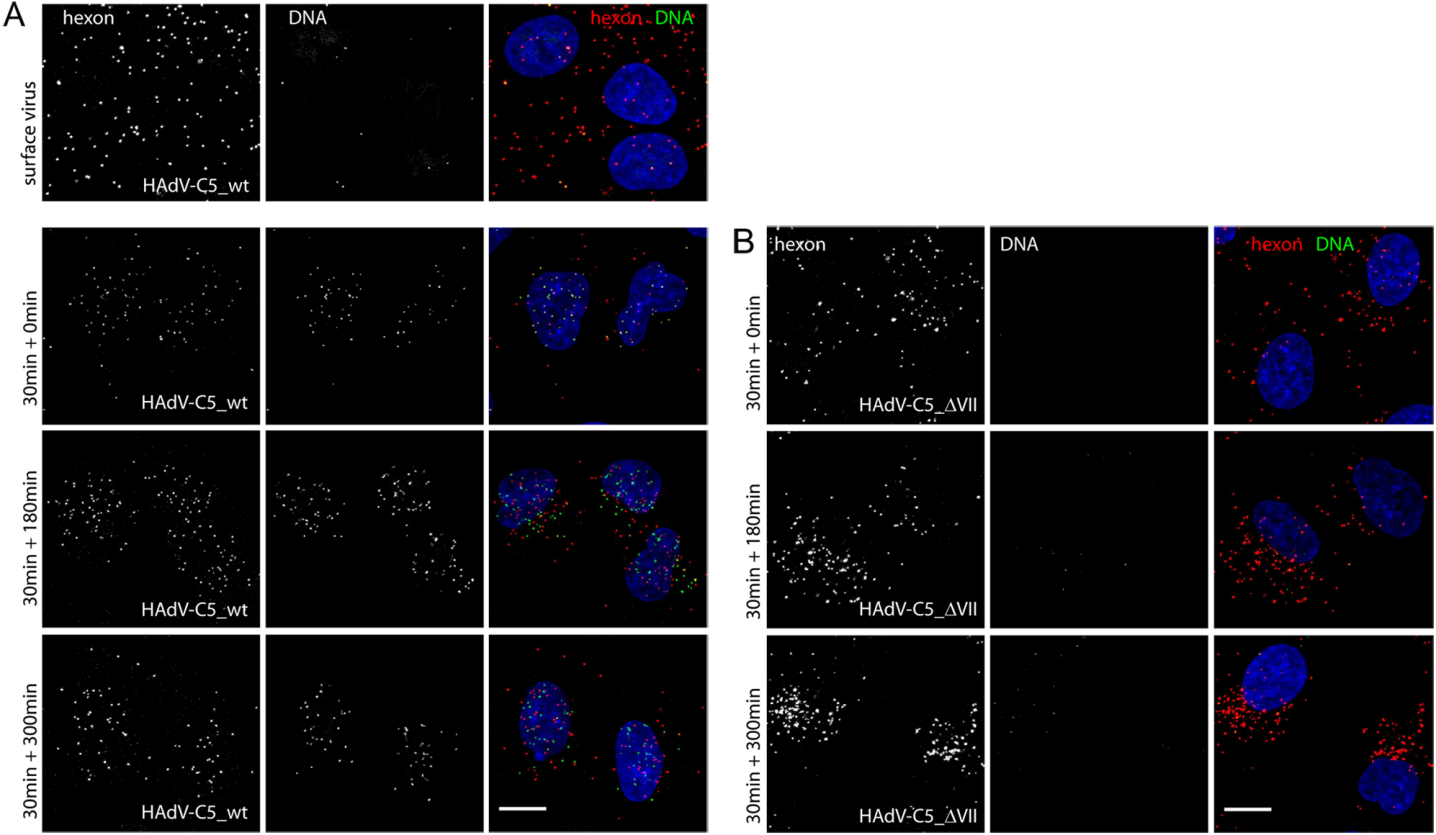
Subcellular localization of VII^−^virus particles analyzed by confocal microscopy. A549 cells were infected with EdC-labeled (see Supplemental Information) Ad5-WT at 4^°^C for 1 hour (A, surface virus) or with Ad5-WT (B, HAdV-C5_wt) or VII^−^virus particles (C, HAdV-C5_ΔVII) at 37^°^C for 30 min. Cells were subsequently incubated for 0 min (30min + 0min), 3 hours (30min + 180min), or 5 hours (30min + 300min). Cells were processed for IF using antibodies directed against intact or partly fragmented Ad5 capsids (hexon, left column, white dots) followed by Click reactions to crosslink Alexa Fluor 488-azide to EdC-labeled viral DNA (center column, DNA, white dots). Panels in the right column represent merged, color images including nuclear DAPI staining. The white bar represents 10μM.

We analyzed the subcellular localization of Ad5 particles that contain or lack protein VII by IF and transmission electron microscopy (TEM). Wild-type Ad5 and Ad-VII-loxP particles produced in Cre-expressing cells (VII^–^) were prepared containing viral genomes labeled with the ethynyl-modified nucleoside EdC. Copper-catalyzed azide-alkyne cycloaddition (Click) reactions allow visualization of single viral genomes within infected cells using Alexa Fluor 488-azide [48]. Cells were infected with EdC-labeled virions, and the major capsid protein hexon was visualized by IF using a specific antibody and viral genomes were visualized following Click reactions (Fig 6). Infections were performed either for 1 hour at 4^°^C to visualize cell surface-bound virus (Fig 6A, top row), or for 30 minutes at 37^°^C, after which unbound virus was washed away and the incubation was continued at 37^°^C for 0 minutes, 3 hours, or 5 hours to visualize internalized virus (Fig 6A and 6B). Virion attachment at the cell surface was readily detected with wild-type Ad5 using anti-hexon antibody but little viral DNA was evident due to protection by the intact capsid [49]. Immediately after 37^°^C infection with wild-type Ad5 (30min + 0min, HAdV-C5_wt), the viral DNA and capsids/capsid remnants were found in both nuclear and cytoplasmic areas. By 3.5–5.5 hpi (30min + 180min, 30min + 300min), wild-type Ad5 capsids and viral DNA were concentrated over the nuclear area marked by DAPI staining. In contrast, the VII^−^mutant virus capsids were largely excluded from within the nuclear area and concentrated in the perinuclear region with time (Fig 6B, HAdV-C5_ΔVII). Due to technical limitations, VII^−^virions were not well detected with EdC within cells, although these virions contained EdC-labeled genomes, as shown by single virus analyses of heat-disrupted particles bound on polylysine-coated coverslips (S3 Fig). VII^−^viral genomes that were evident in infected cells were located outside the nuclei. The localization of VII^−^virions in the perinuclear region is reminiscent of the phenotype observed with *ts*1 virions defective in AVP activity where virions do not efficiently escape the endosome [50–52]. It is possible that incoming VII^-^ virion DNA was not well detected since the VII^-^ virions did not open up their capsids, akin to *ts*1 virions which do not lyse the endosmal membrane [49, 53].

**Fig 7:**
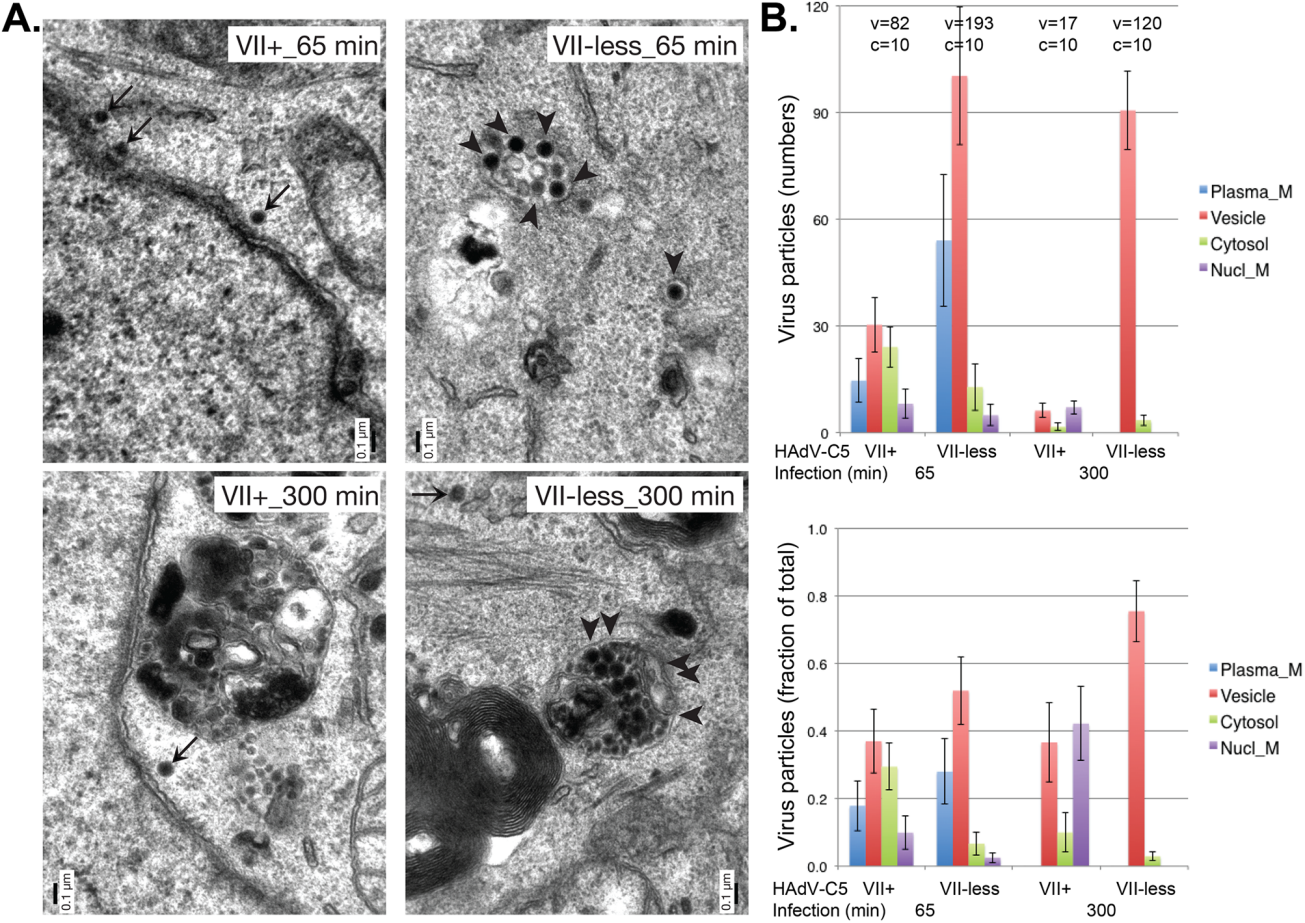
Subcellular localization of VII^−^virus particles analyzed by transmission electron microscopy. A549 cells were infected with VII^+^ or VII^−^virus particles at 37^°^C for 60 min, washed in warm virus-free medium, further incubated at 37^°^C for 5 or 240 min, fixed in glutaraldehyde, and processed for Epon embedding and thin section EM analysis. (A) Representative images of virions in the cytosol or the nuclear membrane (arrows), and in vesicles (arrowheads). (B) Quantification of virions at the plasma membrane (blue bars), in vesicles (red), the cytosol (green), and on the nuclear membrane (violet). The Top panel represents the raw data, including the number of virions (v) and cells (c) for each condition. The Bottom panel shows the normalized data as a fraction of the total number of virions for each condition. Note the enrichment of VII-less virions in multi-vesicular structures, and the strong reduction in wild-type virions 300 min post-infection, indicative of capsid disassembly [49, 88, 89]. The black bar in (A) represents 0.1μm.

The localization of wild-type Ad5 (VII^+^) and VII^−^virions was visualized by TEM at different times after infection (Fig 7). Cells were infected for 1 hour at 37^°^C, washed, and further incubated for 5 minutes or 4 hours at 37^°^C. Incoming virions were visualized by TEM in multiple sections for each condition and scored to be on the plasma membrane, in endosomes, in the cytosol, and on the nuclear membrane. Representative images are shown in Fig 7A, and the data are quantified in Fig 7B. The number of cells (c) and virus particles (v) analyzed for each condition are indicated in the graphs shown in Fig 7B, left, and these results are normalized to the total number of particles analyzed under each condition in Fig 7B, right (total set at 1). The most striking difference between infection with wild-type Ad5 (VII^+^) and VII^−^virions was the disappearance of virus particles with wild-type Ad5 over time by a factor of ~5 in contrast to a small decrease in the number of VII^−^virus particles. The second most striking difference between the VII^+^ and VII^−^virions was the significant clustering of VII^−^virions in cytoplasmic vesicles. This is most drastically illustrated at the 5.5 hour time point, when 10 wild-type virions were found in endosomes (vesicle), contrasting to 115 VII^−^virions in vesicles, including multivesicular eno-lysosomal vesicles. At this time, 5 VII^−^virions were in the cytosol and none were on the plasma membrane or the nuclear membrane. At this time point, 4 wild-type virions were still on the nuclear membrane and 3 were in the cytosol, but most particles were disassembled and could not be recognized as virions. These results illustrate that VII^−^virions do not efficiently escape from endosomes, and that this likely is the reason for the poor localization on the nuclear membrane and the greatly reduced nuclear activity (early gene transcription, viral DNA replication) of VII^−^virions.

## Discussion

The Ad major core protein VII is a histone-like protein that condenses DNA *in vitro* and *in vivo* and assembles viral DNA into a nucleosome-like structure [23–28], although the exact conformation of Ad chromatin within the virion remains elusive [29]. It has been widely assumed that protein VII is required to condense viral DNA within the capsid [29]. Here, we show that the opposite is true. Ad core protein VII is not required for virus assembly or packaging of viral DNA into virions. Virus particles that lack protein VII are stable and contain the normal composition of virion proteins. No change in the amount of core protein V within VII^−^virus particles was observed (Fig 2). These results have important implications for the Ad assembly mechanism and for the role of protein VII during infection. Ad genome packaging is thought to follow the paradigm of dsDNA bacteriophage where viral DNA is inserted into a preformed empty capsid, the prohead [37]. It was not previously known if Ad packages naked viral DNA like dsDNA phage and herpesviruses or a DNA-protein complex containing viral DNA associated with core proteins. It seems very unlikely that Ad evolved a packaging machinery that could accommodate both mechanisms. The process of packaging naked DNA as observed with dsDNA phage involves direct interaction of the packaging motor with the DNA backbone [39], and the interaction of Ad DNA with basic core proteins would seem likely to preclude such interaction. Since viral DNA was efficiently encapsidated in the absence of protein VII, we believe that this strongly supports the model that Ad packages naked DNA, followed by, or in concert with but separate from, the packaging of core proteins. Ads in the mammalian Atadenovirus family do not encode core protein V [54] virtually eliminating a role for this protein in the packaging mechanism. Very little is known about Ad protein X/μ [55], and we have not addressed the potential role of this core protein in the Ad assembly process.

The virion population from the VII-floxed Ad5 grown in the Cre-expressing cells contained a small proportion of unfloxed particles which gave rise to protein VII expression and infection (Fig 3). The particles from the VII-floxed Ad5 entered the cell but were largely defective at endosomal escape (Figs 6 and 7), which resulted in a dramatic decrease in viral early gene expression and viral DNA replication (Fig 4). This defect could not be complemented in *trans* by coinfection with wild-type Ad5 (S2 Fig). Viral DNA isolated from VII^−^virus particles was fully functional when delivered by transfection (Fig 5), but not infection.

The process of Ad entry into the cell and escape from the endosome has been elegantly and extensively studied [56, 57]. Following engagement of Ad5 fiber protein with the primary coxsackievirus adenovirus receptor CAR and secondary binding of penton base to cellular αvβ3/5 integrins, the Ad capsid is internalized by dynamin- and actin-dependent endocytosis into clathrin-coated endosomes. Acidification of the endosome is not required to promote early steps in virion disassembly. Escape of the partly uncoated Ad particle from the endosome is critically-dependent on Ad protein VI [58]. An amphipathic helix near the N-terminus of protein VI binds to the inner surface of the endosomal membrane, induces positive curvature, and appears to fragment the membrane releasing Ad into the cytoplasm [59]. Protein VI is exposed from incoming virions by mechanical cues from differential movements of the virion receptors CAR and integrins [60], and leads to the activation of lysosomal secretion and an increase of ceramide lipids which is key for the membrane disrupting activity of protein VI [61]. Membrane rupture by protein VI occurs through an amphipathic helix near the N-terminus of protein VI, and leads to the rupture of the endosomal membrane [59], and the clearance of the broken vesicles by an autophagic process [62, 63].

The N-terminal 33 amino acids of pre-VI stably bind to the peri-pentonal hexon proteins on the inner surface of the virion [64, 65]. During Ad assembly, this would orient the configuration of pre-VI within the immature capsid. During virion maturation, AVP cleaves pre-VI at the C-terminus to release an AVP-activating peptide and after amino acid 33 to release the remainder of the protein [46]. It is possible, although not very likely, that the failure of capsids that lack protein VII to escape from endosomes may reflect the lack of N-terminal processing of pre-VI observed with the VII^−^mutant virus (Fig 2). We note that non-processed pre-VI displays full membrane lytic activity using *in vitro* assays [47, 59, 66]. An Ad5 site-specific pre-VI mutant that has reduced N-terminal processing by AVP only displays an ~4-fold decrease in infectivity compared to wild-type Ad5 [47]. Thus, the hypothesis that the pVII^−^mutant virus displays an endosome escape defect due to lack of pre-VI N-terminal processing remains unlikely.

Perhaps the defect of the protein VII^−^virions relates to physical changes in the Ad capsid that occur during virion maturation, and such changes are altered in the VII mutant virus infections. Biophysical analyses of the Ad5 core using wild-type Ad5 and *ts*1 grown at the restrictive temperature demonstrated that the Ad core decondenses during proteolytic maturation of the virion resulting in increased internal pressure [67]. This process has been proposed to facilitate virion disassembly during the early stages of infection [67, 68]. *ts*1 capsids produced at the restrictive temperature are highly stable compared to wild-type Ad5 and fail to release capsid vertex proteins even in harsh chemical conditions [69]. Remarkably, the shell of *ts*1 particles is softer than the shell of wild-type virions [69, 70]. Structural studies have shown that this process relates to altered interactions between core components and the internal faces of viral vertex proteins [71]. We propose that these changes in the physical structure of the Ad capsid may not occur properly in the absence of protein VII. Protein VII represents ~10% of the total mass of the Ad particle, and it is possible that the absence of this protein within the capsid would reduce the internal capsid pressure. It will be interesting to test this hypothesis using biophysical approaches. Finally, we cannot exclude the possibility that patches of positively charged residues on viral proteins in the inner surface of the virion unnaturally interact with viral DNA in the absence of protein VII and this interferes with virion disassembly and endosome escape.

VII^−^capsids displayed a pronounced defect in N-terminal pVI processing. AVP proteolytically cleaves Ad proteins pre-IIIa, pre-VI, pre-VII, pre-VIII, pre-X, L1-52/55K, and pre-TP [46, 72]. Pre-IIIa cleavage results in a very minor change in mobility in SDS-PAGE and would not have been detected in our assays. Pre-VI and pre-VIII C-terminal cleavages, and L1-52/55K processing, occurred normally with the VII^−^mutant (Fig 2). The effect of protein VII loss on pre-VI N-terminal cleavage appears to be specific, although reagents are not available to analyze pre-X processing and pre-TP cleavage was not evaluated. It is not clear how pre-VII/VII may affect the activity of AVP, but both interact with viral DNA, and our results suggest that pre-VII/VII influence cleavage events by AVP. AVP slides along DNA within the virion to locate and process substrates [73, 74]. These results suggest that protein VII may influence this process and alter AVP activity and/or specificity. Future studies will be required to clarify the effect of pVII on Ad viron maturation.

In conclusion, Ad core protein VII is not required for virion assembly or viral genome encapsidation. Thus, pre-VII/VII is not required for condensation of Ad DNA within the capsid. The latter result is unexpected. Relative to the length of the Ad genome, the Ad capsid is large compared to some other dsDNA viruses. For example, the Ad capsid diameter is 90–100 nm to accommodate a genome of ~36 kbp. The herpes simplex virus capsid is 100–120 nm to accommodate a genome of ~150 kbp. Similarly, the dsDNA phage T4 capsid is ~85 x 120 nm to accommodate a genome of ~170 kbp. With herpesviruses and dsDNA phages, naked viral DNA is highly condensed within the capsid [39]. The relatively large Ad capsid, as well as the results presented here, suggests that Ad DNA is not as highly condensed within the virion as in case of herpesviruses or phages, perhaps because of a relatively large virion size:genome size ratio. Our results also support the conclusion that Ad likely packages naked DNA, like larger dsDNA viruses. The packaging size limit for Ad5 is ~105% of the full-length genome [75] showing a restriction to capsid capacity. If viral DNA is packaged separately from core proteins, it is an interesting conundrum how a limit for genome size is imposed if there is still space within the virion to subsequently package core proteins. Ad core proteins are not found in empty virions [76], although it is not clear if these capsids are a true assembly intermediate [37]. Light, intermediate capsids observed with Ad5 *ts*369 at the restrictive temperature, which likely do represent a *bona fide* assembly intermediate, contain part of the Ad genome but lack core proteins [42], once again indicating the viral DNA encapsidation precedes core protein insertion. An alternative view of Ad assembly is a concerted mechanism by which the capsid is assembled around the viral DNA-protein core [45]. Our results are consistent with this mechanism of Ad assembly and show that protein VII is not required if this assembly process occurs. This study demonstrates that Ad core protein VII plays an entirely unexpected role during Ad infection, and is required for escape of the virion from the endosome and for full processing of capsid proteins by AVP. It will prove very interesting to determine how protein pre-VII/VII affects capsid pressurization during virion maturation, and if changes in this process underlie the observed phenotype with the VII^−^virions.

## Materials and methods

### Cells

HEK-293 (ATCC), HeLa (ATCC), and A549 cells (ATCC) were maintained in Dulbecco’s modified Eagle’s medium supplemented with 10% bovine calf serum (HyClone), penicillin, and streptomycin. The Cre66 cell line, a Cre recombinase expressing cell line derived from HEK-293 cells (a gift from Dr. Stefan Kochaneck, University Ulm, Germany), was maintained in Dulbecco’s modified Eagle’s medium supplemented with 10% Fetalclone III serum (HyClone), penicillin, streptomycin, and 0.25 mg/ml of Geneticin (Life Technologies). Cre-expressing A549 cells were produced by lentivirus transduction using Cre-IRES-PuroR and maintained in the above mentioned medium supplemented with 8 μg/ml puromycin. Cre-IRES-PuroR was a gift from Darrell Kotton (Addgene plasmid #30205 [77]).

### Viruses and infections

Wild-type adenovirus 5 (Ad5-WT) was derived from the plasmid clone pTG3602 [78] by restriction digestion with PacI and transfection of DNA into cells. Ad2*ts*1 was previously described [23]. The Ad5-VII-loxP virus was generated in the background of pTG3602 in the following way. A subgenomic clone of Ad5 from nucleotides (nt) 12,290–22,340 served as an intermediate vector for the introduction of loxP sites flanking the pVII open reading frame by conventional PCR cloning. The 5’ loxP site was introduced at nt 15,875, three nucleotides upstream of the pVII initiation codon and three nucleotides downstream of the protein III (penton) stop codon. The 3’ loxP site was introduced at nt 16,475, immediately follows the stop codon for pVII. LoxP-containing Ad DNA was recombined with pTG3602 that had been digested with PmeI and AsiSI restriction enzymes, as described [78]. Following confirmation of clones by nucleotide sequence analysis, pTG3602-VII-loxP was digested with PacI, transfected into HEK-293 cells (ATCC), and plaque assays performed. Two independent plaques were amplified and the introduction of the loxP sites was confirmed; these viruses were named Ad5-VII-loxP-5 and Ad5-VII-loxP-11. Stock lysates were generated, titered by plaque assay, and virus particles purified using cesium chloride equilibrium gradient centrifugation, as described [79].

Virus infections were performed for 1 h at 37^°^C, as described [79], unless otherwise noted. The parental Ad5-VII-loxP-5 and Ad5-VII-loxP-11 viruses were used to infect 293 cells at 5 plaque forming units/cell to yield VII^+^ viruses (VII gene intact) or used to infect Cre66 cells (Stefan Kochanek, University Ulm, Germany) to yield VII^−^mutant viruses (VII gene deleted) and harvested 2–3 days after infection when full cytopathic effect was evident. Efficiencies of recombination (floxing) for VII^−^virus were determined using viral DNA extracted from CsCl-purified virus particles by qPCR utilizing two sets primer pairs: 1) Ad5 nt 44–63 and 280–261 to amplify Ad5 left-end sequences to quantify total viral DNA, and 2) Ad5 nt 16,155–16,173 and 16,333–16,315 to amplify sequences within the VII reading frame to quantify viral DNAs with intact VII gene sequences (primer sequences in Supplemental Information). The relative amount of VII^+^ genomes in the VII^−^virus preparations was calculated as described [80]. Low molecular Hirt DNA and purified viral DNA also was analyzed by Southern blot following digestion with KpnI. Southern blots were probed using a DNA fragment including Ad5 nt 15,658–16,887, as described [79]. HeLa cells (ATCC) were infected with the Ad5-VII-loxP viruses, VII^+^ or VII^–^, at 200 virus particles/cell, unless otherwise noted.

### Antibodies

The following primary antibodies were rabbit polyclonal unless indicated and were used at the following dilutions: anti-hexon, mouse monoclonal 9C12 (University of Iowa Developmental Studies Hybridoma Bank, 1:100; rabbit anti-pVII/VII, 1:2000 (Dr. Daniel Engel, University of Virginia); rabbit anti-V, 1:1000 (Dr. David Matthews, University of Bristol); rabbit anti-penton and rabbit anti-fiber, 1:1000 (Dr. Carl Anderson, Brookhaven National Laboratory); rabbit anti-IIIa, 1:1000, [81]; rabbit anti-VIII, 1:400 (Drs. Ann Tollefson and William Wold, St. Louis University); rabbit anti-AVP, 1:500 (Dr. Maxim Balakirev, CEA-Grenoble); rabbit anti-VI, 1:5000 (Dr. Christopher Wiethoff, Loyola University Chicago); rabbit anti-pVI C-terminal peptide amino acids 240–250, 1:100 and rabbit anti-VIII C-terminal peptide amino acids 214–227, 1:1000, (Maarit Suomalainen and Urs Greber, University Zurich, Switzerland); rabbit anti-IVa2 and anti-L1-52/55K 1:1000 [82]; and mouse anti-α-tubulin monoclonal antibody, Sigma-Aldrich T5192.

### Western blot analyses and silver staining of SDS-polyacrylamide gels

Whole cell extracts (WCE) were prepared by washing cells twice with phosphate buffered saline (PBS) followed by cell resuspension in 2X SB (120mM Tris pH 6.8, 4% sodium dodecyl sulfate (SDS), and 20% glycerol) and incubation at 100^°^C for 10 min. Proteins from virus particles were prepared following ethanol precipitation of CsCl-purified virions [83] by resuspension in 2X SB and boiling. Protein concentrations were determined using the bicinchoninic acid (BCA) protein assay kit (Thermo Scientific). Either 15 μg of WCE or 1.3 μg of protein from purified virus particles were separated on 12.5% or 15% SDS-polyacrylamide gels and transferred overnight to nitrocellulose membranes at 4^°^C. Membranes were blocked in 3% bovine serum albumin in TBS (50mM Tris pH 7.5, 150mM NaCl) for 1 hour. Membranes were treated with primary antibodies for 1 hour at room temperature or overnight at 4^°^C and washed 5 times with TBS containing 0.05% Tween20 for 5 min each at room temperature. Secondary antibodies were diluted 1:5000 in 5% powdered milk in TBS, and membranes were treated for 1 hour at room temperature followed by washes as described above. Two additional washes with TBS minus Tween were done before scanning using an Odyssey system (LiCor). IRDye 800CW-conjugated goat anti-rabbit IgG or IRDye 600CW-conjugated goat anti-mouse IgG (LiCor) were used as secondary antibodies for Western blots. SDS-polyacrylamide gels of proteins from particles were stained with silver nitrate using the method of Dr. Darrick Carter (www.proteinchemist.com).

### qPCR and RT-qPCR analyses

At times post-infection indicated in the text, 1-2x10^6^ infected cells were chilled on ice for 10 min, harvested by scraping, washed twice with PBS, and divided for DNA or RNA isolations. Whole cell DNA was isolated using the DNeasy Blood and Tissue Kit (Qiagen). DNAs were quantified by absorbance at 260_nm_. Whole cell RNA was prepared by lysing cells using the QIAshredder (Qiagen) followed by isolation of RNA by RNeasy Plus Mini Kit (Qiagen). cDNAs were generated by priming with oligo(dT) (NEB) and reverse transcription using SuperScript II RT (Invitrogen) following the manufacturer’s instructions. qPCR was performed using the DyNAmo HS SYBER green qPCR kit (Thermo Scientific) and amplifying using the 7500 Real Time PCR System (Applied Biosystems). Reactions contained either approximately 40-80ng of purified DNA or 1/10^th^ of the cDNA product. Results were analyzed using the 7300 system software (Applied Biosystems). Primer pairs for quantification of Ad5 DNA, the Ad5 VII gene, and the cellular GAPDH gene are listed in S1 Table. Primer pairs for cDNA quantification of Ad5 E1a, E2a, E2B, E4, and cellular GAPDH are listed in S1 Table. Standards for absolute quantification were pTG3602 for Ad5-related sequences and subclones of GAPDH specific for either cDNA or gene quantifications. Levels of DNA were determined by dividing the absolute amount of Ad5 DNA by the absolute amount of GAPDH DNA and are graphed on a log scale. Relative levels of Ad early transcripts were calculated using the method of Pfaffl [80] using the absolute amount obtained for viral early transcripts divided by the absolute amount of GAPDH transcripts.

### Immunofluorescence and transmission electron microscopy

For DBP and pVII immunofluorescence microscopy, A549 or HeLa cells grown on glass coverslips were harvested at 24 hours post-infection and processed for immunofluorescence as described [19] using mouse anti-DBP monoclonal antibodies A6-1 and B6-8 [84] and rabbit anti-pVII antibody [31]. Images were captured and analyzed using a Zeiss Axiovert 200M digital deconvolution microscope with AxioVision 4.8.2 SP3 software.

For copper(I)-catalyzed azide alkyne cycloaddition staining and immunofluorescence microscopy, cells grown on glass coverslips were fixed at the times indicated in the text with 3% paraformaldehyde for 15 min, quenched with 25 mM ammonium chloride, permeabilized with 0.5% Triton X-100 at room temperature for 5 min, and labeled with mouse anti-hexon monoclonal antibody 9C12 (University of Iowa Developmental Studies Hybridoma Bank) followed by anti-mouse AlexaFluor 594-conjugated secondary antibody. Samples were stained with freshly prepared click staining mix containing 10 μM AlexaFluor 488-azide, 1 mM CuSO4, and 10 mM sodium ascorbate in PBS in the presence of 1 mM THPTA, and 10 mM amino-guanidine (AG), for protection against oxidative damage, at RT for 2 hr in the dark. Samples were stained with 4′,6-diamidino-2-phenylindole (DAPI, Molecular Probes, Leiden) for total DNA, embedded in DAKO medium (Dako Schweiz AG, Baar) for imaging by confocal microscopy. Fluorescence images were recorded on Leica SP5 confocal laser scanning microscope or Zeiss LSM510 Meta confocal system. Images shown represent maximum projections of confocal stacks.

Samples were processed for transmission electron microscopy (TEM) as previously described [85–87]. In brief, cells grown on glass coverslips were fixed in 1.5% glutaraldehyde–2% formaldehyde, 0.1M sodium cacodylate pH 7.4 for 60 min, followed by post-fixation in 1% OsO_4_ and 1.5% potassium-ferricyanide in deionized water at room temperature for 1 hour, several washes in in 0.1M sodium cacodylate and contrasting 1% tannic acid in 0.05M sodium cacodylate, followed by a 5 min incubation in 1% sodium sulfate in 0.05M sodium cacodylate. The samples were rinsed in deionized water for 5 min, stained with 2% uranyl-acetate overnight, dehydrated with acetone, embedded in Epon, ultrathin-sectioned, and analyzed in a Zeiss EM10 equipped with an advanced interline technology CCD camera Erlangshen ES500W-782 (Gatan GmbH, Munich, Germany). The number of viruses at the plasma membrane, in endosomes, in the cytosol, and at the nuclear membrane was determined by manual counting.

### Transfection of DNAs isolated from virus particles

DNAs from CsCl-purified Ad particles were obtained by ethanol precipitation followed by digestion with proteinase K in the presence of 0.5% SDS, several phenol/chloroform extractions, and ethanol precipitation. DNA was isolated from two different VII^-^ virus particle preparations, from VII^+^ virus particles, and from Ad5-WT virus particles. HeLa cells were transfected with viral DNAs and pcDNA3-EGFP using Lipofectamine 2000 (Invitrogen) and cells were harvested 48 hours post-transfection. DNAs and RNAs were isolated as described above. DNAs were digested with DpnI to fragment input, transfected DNA. DNA and RNA were quantified by qPCR and RT-qPCR, respectively. Primer pairs for DpnI-digested total DNA were Ad5 nt 882–901 and 1052–1033 and primer pairs for cDNA were within the E1a gene.

### Statistical analysis

All numerical values represent mean ± sd. Each experiment was done in three replicates, and a representative replicate is shown for each blot. Statistical significance of the differences was calculated using student’s t-test.

## Supporting information

S1 Fig Analysis of viral DNA in virions that lack protein VII and virus coinfections. Viral DNA was isolated from the virions shown in Fig 2A and analyzed. (A) Viral DNA was analyzed for floxing efficiency as described in Fig 1B. Lane 1, MW markers. Lanes 2–3, DNA from two separate virus preparations of Ad5-VII-loxP grown in 293 cells that express Cre recombinase, lanes 4–5, DNA from two separate virus preparations of Ad5-VII-loxP grown in 293 cells, lanes 6–9, quantification standards. U, unfloxed pVII gene; F, floxed pVII gene. (B) Viral DNAs prepared from purified virions were digested with KpnI and analyzed by Southern blot using a whole Ad5 genome probe. Lane 1, markers. Lanes 2–5, Ad5-VII-loxP viruses grown 293 cells that expression Cre recombinase (lanes 2, 3, virus isolates 5 and 11) or 293 cells (lanes 4, 5, virus isolates 5 and 11). U and F correspond to Ad5 left-end KpnI restriction fragments that are unfloxed or floxed, respectively. (C) Silver stain analysis of the protein composition of CsCl-purified virions isolated from 293 cells infected with wild-type Ad5 (WT) or the Ad5-VII-loxP virus (VII^+^) or 293 cells expressing Cre recombinase (VII^–^) infected with the Ad5-VII-loxP virus, as described in Fig 2.
(TIF)

S2 Fig Coinfection of VII−virions with wild-type Ad5 does not rescue the mutant DNA replication defect. HeLa cells were coinfected with Ad5-WT and Ad5-VII-loxP virus grown in 293 cells (VII+) or 293-Cre cells (VII–). Viral DNAs were isolated at 3 and 24 hours post-infection and quantified by qPCR using virus-specific primer pairs (see S1 Table).
(TIF)

S3 Fig Detection of virus genomes in single virus particles bound on coverslips. EdC-labeled wild-type (wt) or VII^−^virus particles were incubated at room temperature or 55°C, respectively, for 10 min in PBS prior to binding to polylysine-coated glass coverslips. The samples were fixed and stained with 9C12 anti-hexon antibody and anti-mouse AlexaFluor 594-conjugated secondary antibody, followed by Click-reaction with azide-AlexaFluor 488 for detection of the viral genomes. Imaging of the samples was done with a Leica SP5 confocal microscope. **A.** VII^−^virus particles yield lower Click-signal than wild-type particles. Viral DNA is pseudo-colored green and Hexon red, scale bar = 2 μm. At present it is unclear whether the lower EdC-signal from VII^−^particles is due to lower incorporation of EdC into the viral DNA or whether the absence of protein VII imposes a conformation on the genome that is not compatible with efficient Click-detection. The VII^−^virus particles were heat-disrupted at 55°C prior to staining, whereas the wild-type particles were only incubated at room temperature, and this difference explains the apparent separation of DNA and Hexon signals in the VII^−^virus particles. **B.** The lower DNA signal from VII^−^virus particles is not due to lack of genome incorporation into VII^−^particles. In both A and B the Click-signal was detected with a sensitive Hybrid detector (HyD, standard mode), but in B the image acquisition was done with a HyD gain about two-fold higher than in A, and this yielded a readily detectable DNA signal even for the VII^−^particles, whereas no signal was detected from control wild-type particles produced in the absence of EdC labeling.
(TIF)

S1 Table Oligonucleotides. The Table lists the oligonucleotides used for PCR, qPCR, and RT-qPCR.
(DOCX)
