# Supplementary figures and images for: The adenovirus major core protein VII is dispensable for virion assembly but is essential for lytic infection
Source: PLoS Pathog. 2017 Jun 19;13(6):e1006455. doi: 10.1371/journal.ppat.1006455 (PMC5491326; doi:10.1371/journal.ppat.1006455)

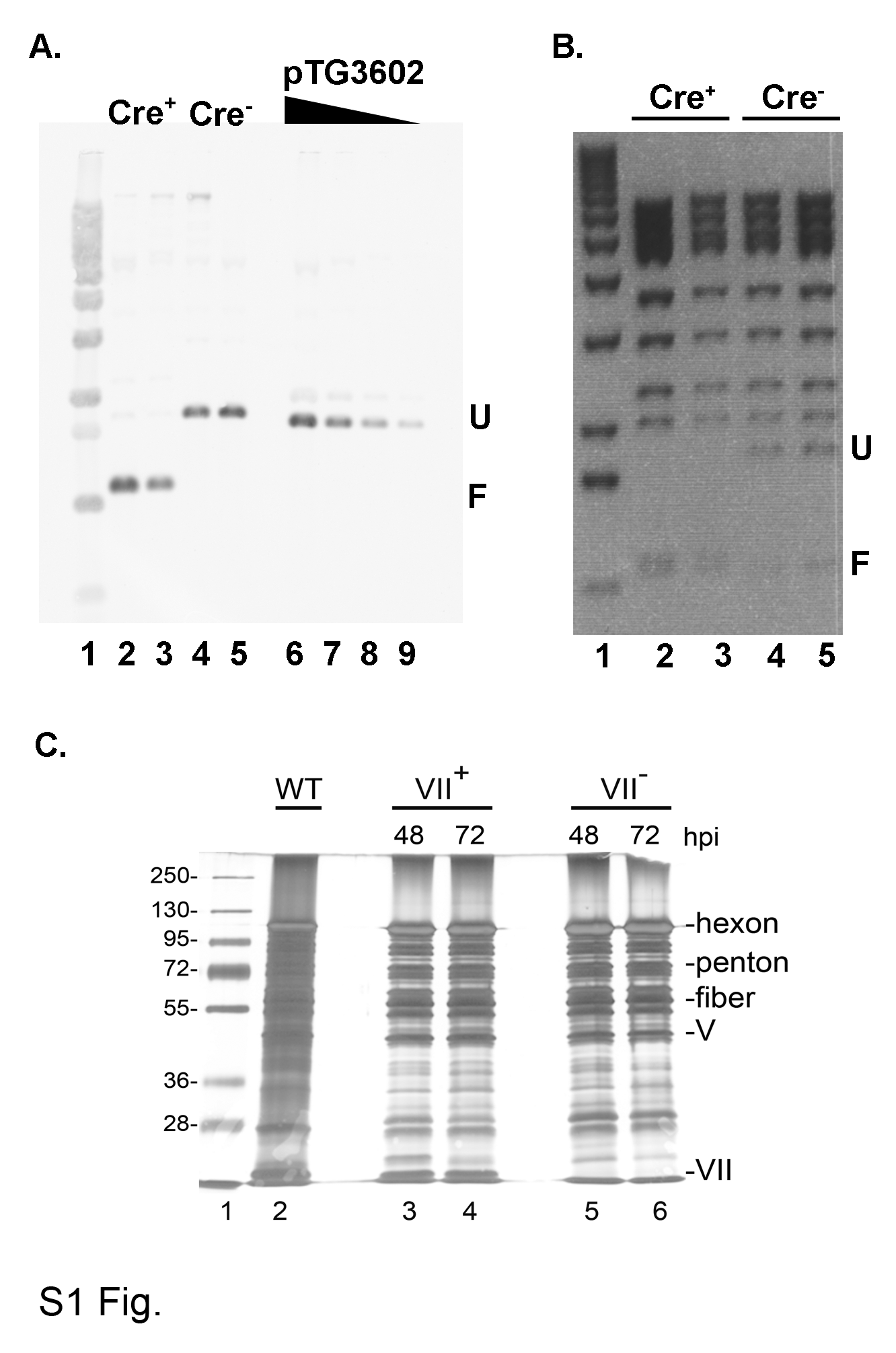

Supplement: S1 Fig — Viral DNA was isolated from the virions shown in Fig 2A and analyzed. (A) Viral DNA was analyzed for floxing efficiency as described in Fig 1B. Lane 1, MW markers. Lanes 2–3, DNA from two separate virus preparations of Ad5-VII-loxP grown in 293 cells that express Cre recombinase, lanes 4–5, DNA from two separate virus preparations of Ad5-VII-loxP grown in 293 cells, lanes 6–9, quantification standards. U, unfloxed pVII gene; F, floxed pVII gene. (B) Viral DNAs prepared from purified virions were digested with KpnI and analyzed by Southern blot using a whole Ad5 genome probe. Lane 1, markers. Lanes 2–5, Ad5-VII-loxP viruses grown 293 cells that expression Cre recombinase (lanes 2, 3, virus isolates 5 and 11) or 293 cells (lanes 4, 5, virus isolates 5 and 11). U and F correspond to Ad5 left-end KpnI restriction fragments that are unfloxed or floxed, respectively. (C) Silver stain analysis of the protein composition of CsCl-purified virions isolated from 293 cells infected with wild-type Ad5 (WT) or the Ad5-VII-loxP virus (VII+) or 293 cells expressing Cre recombinase (VII–) infected with the Ad5-VII-loxP virus, as described in Fig 2. (TIF) [file ppat.1006455.s001.tif]

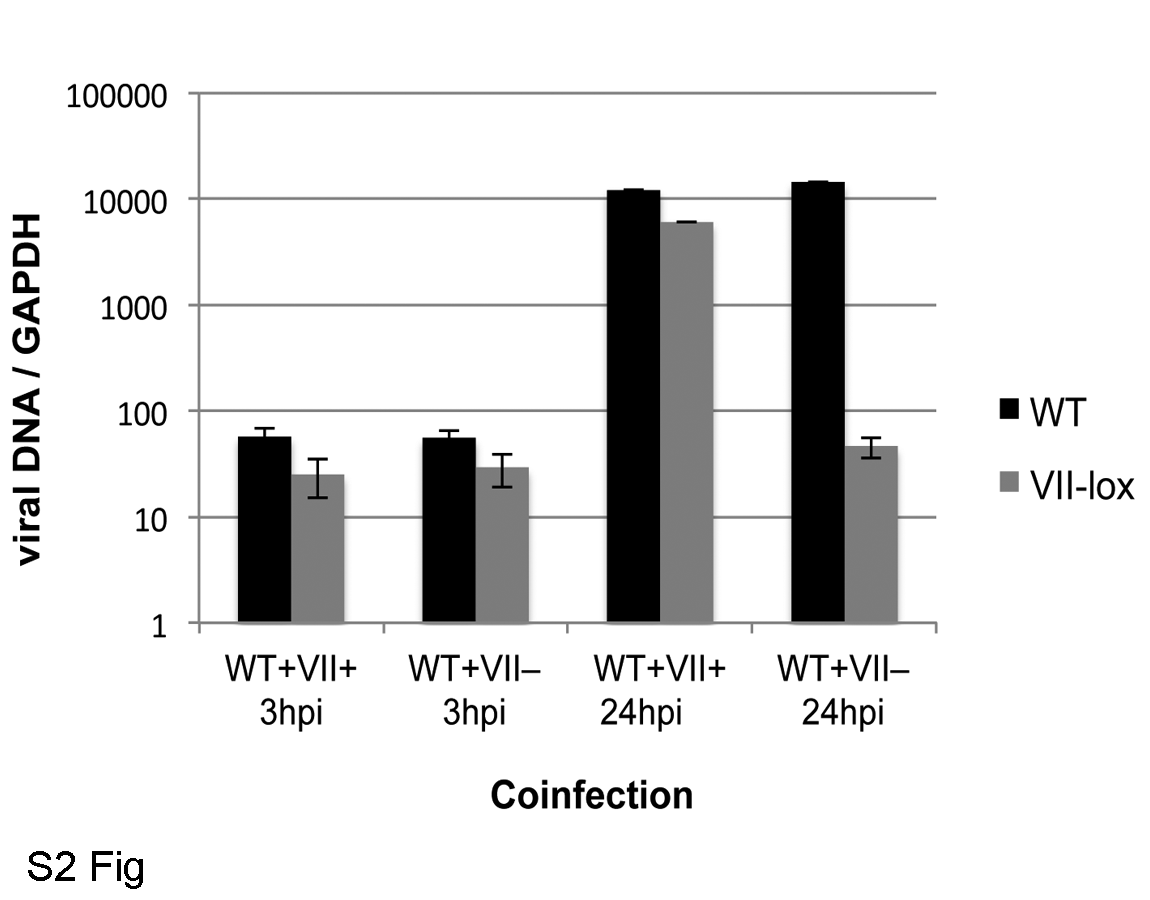

Supplement: S2 Fig — HeLa cells were coinfected with Ad5-WT and Ad5-VII-loxP virus grown in 293 cells (VII+) or 293-Cre cells (VII–). Viral DNAs were isolated at 3 and 24 hours post-infection and quantified by qPCR using virus-specific primer pairs (see S1 Table). (TIF) [file ppat.1006455.s002.tif]

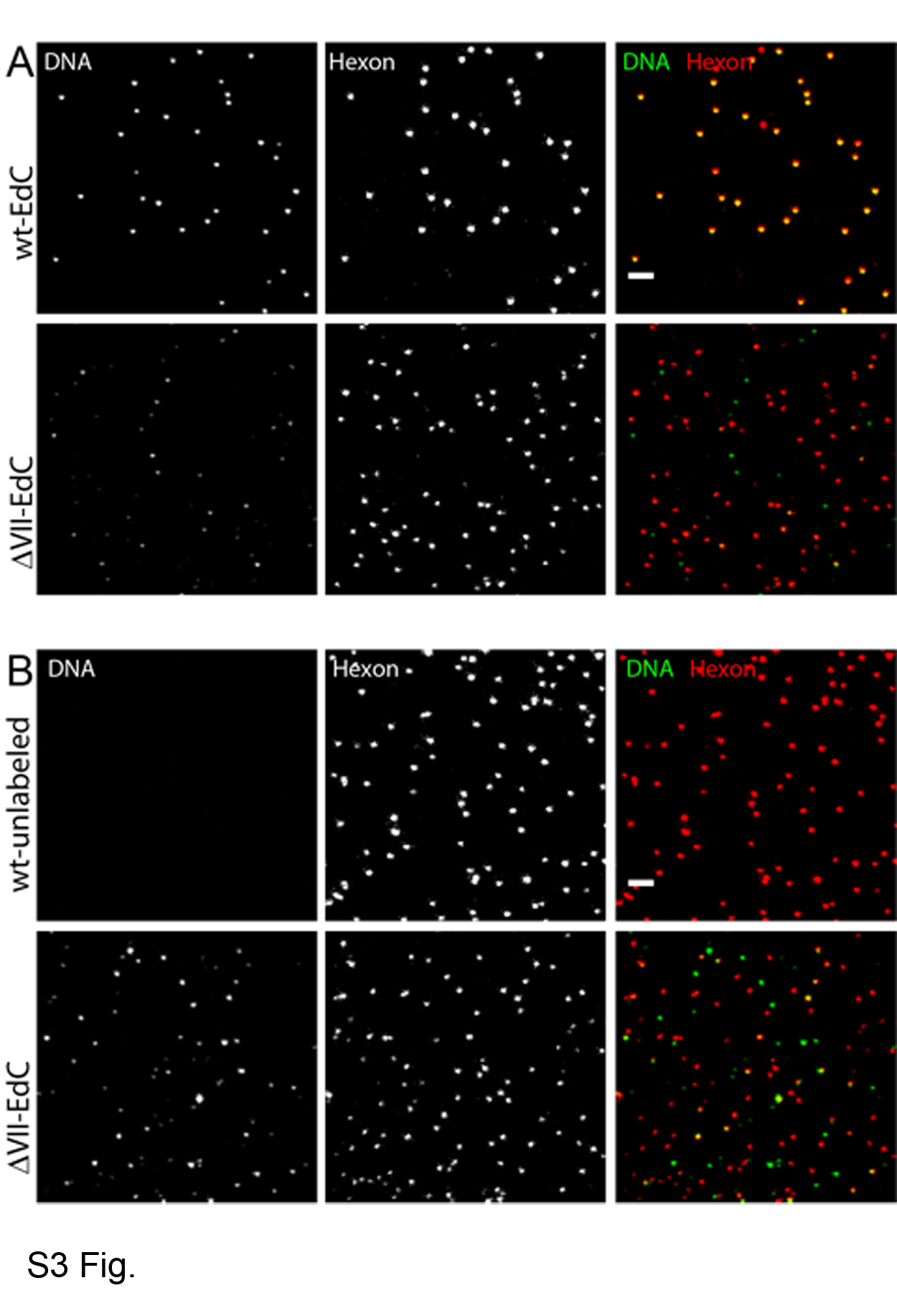

Supplement: S3 Fig — EdC-labeled wild-type (wt) or VII−virus particles were incubated at room temperature or 55°C, respectively, for 10 min in PBS prior to binding to polylysine-coated glass coverslips. The samples were fixed and stained with 9C12 anti-hexon antibody and anti-mouse AlexaFluor 594-conjugated secondary antibody, followed by Click-reaction with azide-AlexaFluor 488 for detection of the viral genomes. Imaging of the samples was done with a Leica SP5 confocal microscope. A. VII−virus particles yield lower Click-signal than wild-type particles. Viral DNA is pseudo-colored green and Hexon red, scale bar = 2 μm. At present it is unclear whether the lower EdC-signal from VII−particles is due to lower incorporation of EdC into the viral DNA or whether the absence of protein VII imposes a conformation on the genome that is not compatible with efficient Click-detection. The VII−virus particles were heat-disrupted at 55°C prior to staining, whereas the wild-type particles were only incubated at room temperature, and this difference explains the apparent separation of DNA and Hexon signals in the VII−virus particles. B. The lower DNA signal from VII−virus particles is not due to lack of genome incorporation into VII−particles. In both A and B the Click-signal was detected with a sensitive Hybrid detector (HyD, standard mode), but in B the image acquisition was done with a HyD gain about two-fold higher than in A, and this yielded a readily detectable DNA signal even for the VII−particles, whereas no signal was detected from control wild-type particles produced in the absence of EdC labeling. (TIF) [file ppat.1006455.s003.tif]
