# Supplementary material for: The adenovirus major core protein VII is dispensable for virion assembly but is essential for lytic infection
Source: PLoS Pathog. 2017 Jun 19;13(6):e1006455. doi: 10.1371/journal.ppat.1006455 (PMC5491326; doi:10.1371/journal.ppat.1006455)
Supplement: S1 Table — The Table lists the oligonucleotides used for PCR, qPCR, and RT-qPCR. (DOCX) [file ppat.1006455.s004.docx]

**S1 Table. Oligonucleotides.**

| **Name** | **Sequence (5’-3’)** | **Notes** |
| --- | --- | --- |
| Ad5 12,071-12,086 | GCACAGCCTGCAAAGG | primers used to insert loxP sites at the 5’ and 3’ ends of the pVII open reading frame by PCR cloning |
| VII+loxP-5-1 | GCCGCACTTTTTGAGCAATAACTTCGTATAATGTATGCTATACGAAGTTATAGCATGTCCATCCTTATAT |  |
| VII+loxP-5-2 | ATATAAGGATGGACATGCTATAACTTCGTATAGCATACATTATACGAAGTTATTGCTCAAAAAGTGCGGC |  |
| VII+loxP-3-1 | CCCCCGCGCAACTAGATAACTTCGTATAATGTATGCTATACGAAGTTATATATTGCAAGAAAAAACTACTTA |  |
| VII+loxP-3-2 | TAAGTAGTTTTTTCTTGCAATATATAACTTCGTATAGCATACATTATACG  AAGTTATCTAGTTGCGCGGGGG |  |
| Ad5 18,253-18,238 | CAGGCCATCTACCACC |  |
| Ad5 nt 44-63 | GCGAAAATGGCCAAATGTTA | Ad5 left-end qPCR |
| Ad5 nt 280-261 | TAATGAGGGGGTGGAGTTTG |  |
| Ad5 nt 16,155-16,173 | GGCGCTATGCTAAAATGAA | Ad5 pVII gene qPCR |
| Ad5 nt 16,333-16,315 | GGGGGCACAGTGACAATA |  |
| gGAPDH-F | CCCCACACACATGCACTTACC | cellular GAPDH genomic sequence qPCR |
| gGAPDH-R | CCTAGTCCCAGGGCTTTGATT |  |
| E1A-F | TCCGGTCCTTCTAACACACC | E1a cDNA RT-qPCR |
| E1A-R | GGCGTTTACAGCTCAAGTCC |  |
| DBP-F | CCGTAGTGGCATCAAAAGGT | E2a cDNA  RT-qPCR |
| DBP-R | GTCTAGCAAGGCCAAGATCG |  |
| E2B-F | CGCGCGTCGAAGTAGTCTAT | E2b cDNA RT-qPCR |
| E2B-R | CGGTGGAAGATGCTACCCTA |  |
| E4ORF6-F | TACCGGGAGGTGGTGAATTA | E4 cDNA RT-qPCR |
| E4ORF6-R | TTCAAAATCCCACAGTGCAA |  |
| cGAPDH-F | ACCCAGAAGACTGTGGATGG | cellular GAPDH cDNA RT-qPCR |
| cGAPDH-R | TTCTAGACGGCAGGTCAGGT |  |
| Ad5 nt 882-901 | GTCCGGTTTCTATGCCAAAC | Ad5 left-end sequences with DpnI restriction sites |
| Ad5 1052-1033 | CCGTATTCCTCCGGTGATAA |  |
| Ad5 15768-15787 | TCCAGCGAGTGACCATTACT | primer pair that only recognizes Ad DNAs with a loxP site at the 5’ end of the VII gene |
| VII-lox-5 | TATAGCATACATTATACGAAGTTATTGCTCAAA |  |
